# Residential Exposure to Outdoor Air Pollution during Pregnancy and Anthropometric Measures at Birth in a Multicenter Cohort in Spain

**DOI:** 10.1289/ehp.1002918

**Published:** 2011-03-23

**Authors:** Marisa Estarlich, Ferran Ballester, Inmaculada Aguilera, Ana Fernández-Somoano, Aitana Lertxundi, Sabrina Llop, Carmen Freire, Adonina Tardón, Mikel Basterrechea, Jordi Sunyer, Carmen Iñiguez

**Affiliations:** 1 Consortium for Research on Epidemiology and Public Health (CIBERESP), Spain; 2 Center for Public Health Research (CSISP), Valencia, Spain; 3 University of Valencia, Valencia, Spain; 4 Center for Research in Environmental Epidemiology (CREAL), Barcelona, Spain; 5 Hospital del Mar Research Institute (IMIM), Barcelona, Spain; 6 Department of Preventive Medicine, University of Oviedo, Asturias, Spain; 7 Public Health Division of Gipuzkoa, Basque Government, Gipuzkoa, Spain; 8 Department of Preventive Medicine and Public Health, (EHU-UPV), University of the Basque Country, Gipuzkoa, Spain.; 9 Laboratory of Medical Investigations, San Cecilio University Hospital, University of Granada, Granada, Spain; 10 Pompeu Fabra University, Barcelona, Spain

**Keywords:** air pollution, anthropometry, benzene, birth, birth weight, nitrogen dioxide, pregnancy

## Abstract

Background: A growing body of research suggests that prenatal exposure to air pollution may be harmful to fetal development. We assessed the association between exposure to air pollution during pregnancy and anthropometric measures at birth in four areas within the Spanish Children’s Health and Environment (INMA) mother and child cohort study.

Methods: Exposure to ambient nitrogen dioxide (NO_2_) and benzene was estimated for the residence of each woman (*n =* 2,337) for each trimester and for the entire pregnancy. Outcomes included birth weight, length, and head circumference. The association between residential outdoor air pollution exposure and birth outcomes was assessed with linear regression models controlled for potential confounders. We also performed sensitivity analyses for the subset of women who spent more time at home during pregnancy. Finally, we performed a combined analysis with meta-analysis techniques.

Results: In the combined analysis, an increase of 10 µg/m^3^ in NO_2_ exposure during pregnancy was associated with a decrease in birth length of –0.9 mm [95% confidence interval (CI), –1.8 to –0.1 mm]. For the subset of women who spent ≥ 15 hr/day at home, the association was stronger (–0.16 mm; 95% CI, –0.27 to –0.04). For this same subset of women, a reduction of 22 g in birth weight was associated with each 10-µg/m^3^ increase in NO_2_ exposure in the second trimester (95% CI, –45.3 to 1.9). We observed no significant relationship between benzene levels and birth outcomes.

Conclusions: NO_2_ exposure was associated with reductions in both length and weight at birth. This association was clearer for the subset of women who spent more time at home.

In the last decade, a growing body of research has found associations between prenatal exposure to air pollution and adverse birth outcomes, including low birth weight and reduced anthropometric measures. Although most studies have noted certain associations between different indicators of air pollution and birth size, the results are inconclusive, as indicated in reviews on the topic (Glinianaia et al. 2004; Lacasaña et al. 2005; Ritz and Wilhelm 2008; Slama et al. 2008; Šrám et al. 2005; Woodruff et al. 2009). Such reviews emphasize the need to examine several methodologic aspects in greater detail, such as the collection of detailed data concerning the covariates, the exposure measurement process, and the assessment of critical exposure windows. Cohort studies starting at the beginning of pregnancy are adequate for dealing with all of these issues.

With regard to exposure assessment, several techniques can be used to obtain air pollution estimates at unmonitored sites, including the homes of pregnant women in a cohort. The development of land use regression (LUR) models, which use regression to map air pollution using variables such as land use, traffic density, population, and other geographic variables as predictors (Aguilera et al. 2008; Iñiguez et al. 2009), has been especially helpful, because these models can detect small area variations in air pollution levels more accurately than other interpolation methods (Ryan and Lemasters 2007). In addition, they allow for the introduction of temporal variability into the monitored network. Finally, the time–activity patterns and changes of residence of subjects during pregnancy must be taken into account (Fell et al. 2004; Nethery et al. 2009; Ritz and Wilhelm 2008) to reduce exposure misclassification.

Another problem addressed is the critical exposure windows. Numerous epidemiologic studies suggest that exposure to air pollution in specific periods of pregnancy leads to distinct reproductive outcomes (Glinianaia et al. 2004). However, although some studies have found the effects of air pollution on birth outcomes to be greater earlier in pregnancy, others have identified them as being more harmful later in pregnancy (Ritz and Wilhelm 2008; Woodruff et al. 2009). These inconsistent results reinforce the importance of examining the critical exposure windows during pregnancy in greater detail.

The Spanish Children’s Health and Environment (INMA) study is a network of seven pregnancy/birth cohorts in various areas of Spain with different sociodemographic and environmental patterns (INMA 2011) established to evaluate the role of the environment on fetal development and children’s health (Ribas-Fitó et al. 2006). Previously published studies carried out separately assessed the association between air pollution exposure and certain reproductive outcomes (Aguilera et al. 2009; Ballester et al. 2010). However, because the analysis of a single cohort limits the power of a study to detect differences by pregnancy period or the influence of time–activity patterns during pregnancy, a joint analysis is necessary. We have undertaken such a study to assess residential exposure to outdoor air pollution throughout pregnancy and its relationship to anthropometric measures.

## Materials and Methods

**Figure 1:**
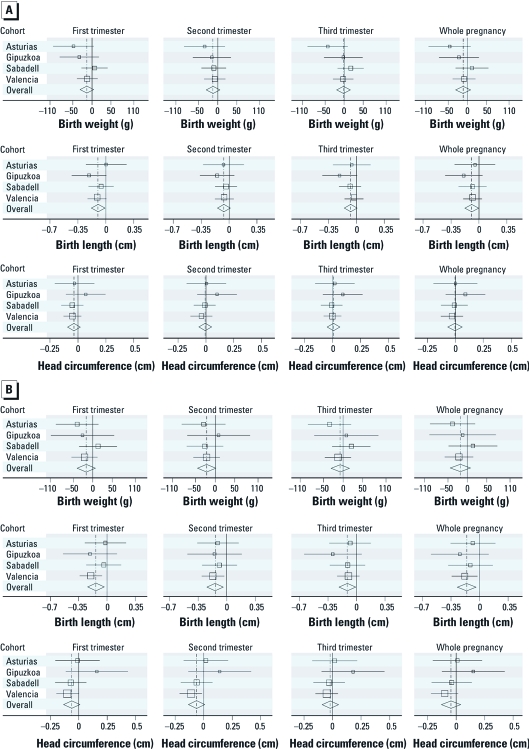
(*A*) Relationship between NO_2_ exposure (micrograms per cubic meter) by trimester of pregnancy and birth outcomes for the entire sample: single-pollutant models. (*B*) Relationship between NO_2_ exposure (micrograms per cubic meter) by trimester of pregnancy and birth outcomes in women who spent ≥ 15 hr/day at home: single-pollutant models.

*Study design and population.* Our study was based on data from the four new INMA project cohorts (Asturias, Gipuzkoa, Sabadell, and Valencia), which had followed the same protocol since the beginning of pregnancy [see Supplemental Material, Table 1 and Figure 1 (http://dx.doi.org/10.1289/ehp.1002918)]. Pregnant women were recruited between November 2003 and February 2008. Subject recruitment and follow-up procedures have been reported elsewhere (Ribas-Fitó et al. 2006). Briefly, the inclusion criteria were age ≥ 16 years, singleton pregnancy, enrollment at 10–13 weeks of gestation, no assisted conception, delivery scheduled at the reference hospital, and no communication handicap. A total of 2,644 eligible pregnant women agreed to participate in the study. All participants signed informed consent forms, and the research protocol was approved by the ethics committees of the various centers involved in the study. From May 2004 to August 2008, after excluding the women who withdrew, were lost to follow-up, or underwent induced or spontaneous abortions or fetal deaths, we monitored a sample of 2,505 women until delivery.

**Table 1:** Anthropometric measures and air pollution levels in the study by cohort (mean ± SD).

Study population										
Variable	Overall (*n *= 2337)	Asturias (*n* = 417)	Gipuzkoa (*n* = 573)	Sabadell (*n* = 563)	Valencia (*n* = 784)	*p*-Value*ª*
Anthropometric measures												
Weight (g)		3,342 ± 401		3,365 ± 386		3,367 ± 392		3,312 ± 391		3,333 ± 419		0.065
Length (cm)		49.9 ± 1.8		50.1 ± 1.9		49.2 ± 1.7		49.6 ± 1.7		50.5 ± 1.8		< 0.001
HC (cm)		34.4 ± 1.3		34.4 ± 1.3		34.8 ± 1.3		34.3 ± 1.1		34.3 ± 1.3		< 0.001
Levels of air pollution (µg/m^3^)												
NO_2_												
All		29.2 ± 11.1		23.5 ± 6.5		20.1 ± 6.4		31.9 ± 8.6		36.9 ± 11.1		< 0.001
Urban		29.8 ± 11.0		23.8 ± 6.5		20.2 ± 6.4		31.9 ± 8.6		38.3 ± 9.8		
Rural		16.4 ± 4.9		18.0 ± 5.3		17.5 ± 5.9		NA		15.1 ± 3.9		
Benzene												
All		1.6 ± 1.1		2.3 ± 1.3		1.0 ± 0.3		0.81 ± 0.3		2.17 ± 0.6		< 0.001
Urban		1.6 ± 0.9		2.3 ± 1.3		1.0 ± 0.3		0.81 ± 0.3		2.2 ± 0.6		
Rural		1.5 ± 0.7		1.7 ± 1.1		0.9 ± 0.2		NA		1.7 ± 0.6		
NA, not applicable. **a**Analysis of variance *p*-value for the comparison among cohorts.												

*Assessment of air pollution exposure.* A protocol was designed to assess individual exposure to nitrogen dioxide (NO_2_) and benzene as markers of outdoor air pollution. Ambient concentrations were measured with the aid of passive samplers (Radiello, Fundazione Salvatore Maugeri, Padua, Italy) distributed over the study areas according to geographic criteria, taking into account the expected pollution gradients and the distribution of the residences of the women. The samplers remained exposed during various 7-day sampling periods. The methodology has been described in detail elsewhere (Aguilera et al. 2008; Iñiguez et al. 2009); further information is given in Supplemental Material, Table 2 (http://dx.doi.org/10.1289/ehp.1002918).

**Table 2:** Association between individual exposure to ambient NO_2_^*a*^ and benzene^*b*^ during pregnancy and anthropometric measures at birth: meta-analysis results from the four different cohort-specific estimates [β (95% CI)].

Outcome	Exposure	All women (*n* = 2,337)	Women who spent ≥ 15 hr/day at home (*n* = 1,380)
Adjusted one-pollutant model						
Weight (g)		NO_2_		–10.8 (– 31.2 to 9.8)		–17.8 (–44.1 to 8.6)
		Benzene		–4.2 (–34.5 to 26.1)		–7.4 (–42.8 to 27.9)
Length (cm)		NO_2_		–0.09 (–0.18 to –0.01)		–0.16 (–0.27 to –0.04)
		Benzene		–0.01 (–0.14 to 0.13)		–0.02 (–0.18 to 0.15)
HC (cm)		NO_2_		–0.004 (–0.069 to 0.061)		–0.045 (–0.130 to 0.039)
		Benzene		0.03 (–0.07 to 0.13)		0.04 (–0.08 to 0.15)
Adjusted two-pollutant model						
Weight (g)		NO_2_		–16.7 (–59.7 to 26.3)		–17.9 (–54.3 to 18.4)
		Benzene		16.2 (–24.6 to 56.9)		14.2 (–32.1 to 60.5)
Length (cm)		NO_2_		–0.16 (–0.29 to –0.03)		–0.23 (–0.39 to –0.07)
		Benzene		0.16 (–0.03 to 0.35)		0.17 (–0.04 to 0.38)
HC (cm)		NO_2_		–0.003 (–0.097 to 0.090)		–0.057 (–0.172 to 0.059)
		Benzene		0.04 (–0.09 to 0.17)		0.08 (–0.08 to 0.24)
**a**Results for a 10-µg/m^3^ increase in NO_2_. Birth weight (model 1): adjusted for maternal age, maternal prepregnancy weight, maternal height, paternal height, gestational weight gain, parity, cohabitation with the baby’s father, maternal working status, smoking during pregnancy, country of origin, sex of the infant, rural, and season of last menstrual period, type of cooker; birth length: model 1 + maternal social class; birth HC: model 1 + maternal education. **b**Results for a 1-unit increase in log_2_-transformed benzene (micrograms per cubic meter). Birth weight and birth length: adjusted for model 1 covariates + maternal social class; birth HC: model 1 + maternal social class + maternal education.						

LUR was used to predict NO_2_ and benzene levels at unmonitored sites, including outside the residences of the women. Because of the scarcity of geographic information from rural sites in Gipuzkoa and Asturias, women in these cohorts who lived farther than 1 km from a passive sampler were excluded from the present analysis. The model variables for each cohort are described in the Supplemental Material, Table 2 (http://dx.doi.org/10.1289/ehp.1002918).

To calculate individual exposure during the pregnancy of each woman, NO_2_ spatial estimates were temporally adjusted by using the daily NO_2_ levels obtained from the monitoring network stations covering the study area. Some monitoring stations for benzene were running at the same areas, but these had a high percentage of missing data. Thus, data from the monitored air pollutant exhibiting the best correlation in each cohort [Supplemental Material, Table 2 (http://dx.doi.org/10.1289/ehp.1002918)] were used to adjust for seasonal variability, as in previous studies (Aguilera et al. 2009; Slama et al. 2007). The same procedure was used to calculate air pollution exposure for each trimester of pregnancy. Change of residential address during pregnancy was taken into consideration only when women lived at least 2 months of the pregnancy period in the new residence, which occurred in 1–6% of the cases, depending on the cohort.

*Birth outcome assessment.* The outcome variables were birth weight in grams and birth length and head circumference (HC), both in centimeters. Birth weight was measured by the midwife attending the birth, whereas birth length and HC were measured by a nurse when the newborn arrived at the hospital ward within the first 12 hr of life. An early ultrasound of the crown–rump length was also available and was used for gestational dating when the difference with the last menstrual period was ≥ 7 days (12% of the cases). Growth curves for birth weight, length, and HC were fitted to further standardize them to week 40 of gestation using the Box–Cox power exponential method (Rigby and Stasinopoulos 2004) and adjusting by sex and cohort.

*Covariates and potential confounders.* The mothers completed detailed questionnaires about their sociodemographic characteristics, environmental exposures, and lifestyle variables at weeks 12 and 32 of pregnancy. Potential confounders were selected based on previous scientific findings. Confounders included maternal variables [age, height, prepregnancy weight, prepregnancy body mass index (BMI), weight gain, education, working status, socioeconomic status, country of origin (Spain vs. foreign), cohabitation with the father of the baby, smoking, and environmental tobacco exposure], infant sex, paternal height, type of zone (urban vs. rural), and season of last menstrual period. The mother’s rate of weight gain was calculated in kilograms per week during the second and third trimesters of pregnancy and classified as low, medium, or high, depending on the mother’s prepregnancy BMI, as described recently by the Institute of Medicine (IOM) guidelines (2009). Socioeconomic status was divided into three occupational categories according to the current or most recent occupation of the mother (or the father, if the mother had never worked outside the home) on the basis of a widely used Spanish adaptation of the British classification system (Domingo Salvany et al. 2000). Working status (working or not) was assessed in the first and third trimesters. Women were considered to be smokers if they reported smoking at week 32. Exposure to environmental tobacco smoke was assessed as both passive exposure at home and global exposure (either at home, at work, or at leisure). Parity was defined as the number of previous pregnancies that lasted at least 22 weeks; subjects were categorized as women without children or women with one child or more. The type of cooker used was divided into three categories: electric, gas, and other.

*Statistical analysis.* We described NO_2_ and benzene levels by trimester as well as throughout the entire pregnancy and calculated the correlations between them for each cohort. Because of their skewed distribution, benzene levels were log_2_ transformed. We first performed a cohort-specific analysis, using bivariate regression models to determine which parental and pregnancy characteristics were associated with the various birth outcomes. In the multiple analysis, the covariates were retained in the final model if they were related to the outcome [based on likelihood ratio (LR) tests with a *p*-value of < 0.10] or if the effect estimates for the exposure of interest changed by ≥ 10% when they were excluded from the model. Age of the mother, season, and type of zone were included in all models despite their statistical significance. We adjusted generalized additive models to assess the shape of the relationship between birth measurements and NO_2_ and benzene levels (Hastie and Tibshirani 1990). Using this approach, we evaluated the linearity of the association between air pollution levels and reproductive outcomes, comparing linear and nonlinear models (a cubic smoothing spline with 1, 2, and 3 knots) with the aid of graphical examination and the LR test (*p* < 0.05). To account for the possible influence of extreme values, we ran the models both with and without them.

We also performed a sensitivity analysis taking into account the time–activity patterns of the women during pregnancy. We calculated the time spent at home from self-reported information (questionnaire at week 32) and restricted our analysis to women who spent ≥ 15 hr/day at home. Given the importance of exposure to tobacco smoke on fetal growth, we also analyzed this variable, dividing it into three categories: nonsmokers during pregnancy, smokers during the first trimester but not after week 12, and still smoking after week 12.

Finally, we performed a combined analysis using meta-analysis techniques. The combined estimates were obtained through weighted regression in which the weights were the inverse of the local variances, that is, the fixed-effect model. Heterogeneity was quantified with the *I*-squared measure (*I*^2^) (Higgins et al. 2003) under the fixed-effect hypothesis; if heterogeneity was detected (*I*^2^ > 50% or *p* < 0.15), the random-effect model was applied.

## Results

*Study population characteristics and air pollution levels.* Outcomes and exposure variables are described in Table 1. Complete details of the characteristics of the study population are given in Supplemental Material, Table 3 (http://dx.doi.org/10.1289/ehp.1002918). Slight yet statistically significant differences were observed for birth outcomes in the different cohorts. Differences were found for maternal social class, education, working status, exposure to tobacco smoke, and country of origin, with Valencia and Sabadell being the cohorts with the highest percentage of foreigners. Especially noteworthy is the fact that 65% of the non-Spanish women were Latin American. Pollutant levels also varied among cohorts, with NO_2_ levels being lower in Asturias and Gipuzkoa and higher in Sabadell and Valencia (Table 1). In contrast, the highest benzene levels were found in Asturias. Within each cohort, we found differences depending on the type of zone, with higher levels in urban zones than in rural ones. Pollutant levels by trimester presented medium to high correlations (Spearman rho: 0.38–0.78). Residential NO_2_ levels throughout the entire pregnancy correlated well with benzene levels in Gipuzkoa, Sabadell, and Valencia (Spearman rho: 0.7), whereas in Asturias, the correlation between benzene and NO_2_ levels was only moderate (Spearman rho: 0.29).

*Air pollution exposure and anthropometric measures.* Because nonlinear models did not provide a better fit, the relationship between air pollution and anthropometric measures at birth was assessed linearly. Figure 1 shows the estimates of association in each cohort by each pregnancy term, as well as the combined results for the whole sample (Figure 1A) and in women who spent ≥ 15 hr/day at home (Figure 1B). In general, the results by cohort showed no significant heterogeneity in any of the cases (*p*-value for heterogeneity < 0.15 and *I*^2^ > 50%). We obtained an *I*^2^ = 50.3 and *p* = 0.108 for the weight model with NO_2_ adjusted for two pollutants. The results were not affected by the inclusion of extreme values. Combined estimates based on adjusted single-pollutant models for the whole pregnancy showed a significant negative association between NO_2_ levels and birth length. On average, we found that a 10-µg/m^3^ increase in residential NO_2_ exposure throughout the entire pregnancy was significantly associated with a 0.9-mm [95% confidence interval (CI), –0.18 to –0.01] decrease in birth length for all participants (Table 2). An inverse association between NO_2_ exposure and birth weight was also found, with a 10-µg/m^3^ increase in NO_2_ during the whole pregnancy being related to a 10.8-g decrease in birth weight; this relationship was not statistically significant (95% CI, –31.2 to 9.8). The associations between NO_2_ levels and birth outcomes were stronger in the subset of women who spent ≥ 15 hr/day at home, but except for birth length, they lacked statistical significance (Table 2). The estimated NO_2_ coefficients for birth length and weight were greater when benzene exposure was also included in the model. Interestingly, benzene exposure was not significantly associated with any anthropometric measure at birth, in either a single- or a two-pollutant model.

In the analysis by trimester, the associations were likewise strengthened when the analysis was limited to women who spent > 15 hr/day at home (Figure 1B). For birth weight, a pattern of inverse associations appeared during the first and especially during the second trimester. Thus, a 10-µg/m^3^ increase in NO_2_ in the second trimester was associated with a 21.7-g decrease in birth weight (95% CI, –45.3 to 1.9). A significant combined association for birth length was observed in all three trimesters, with the second trimester exhibiting the clearest relationship. For HC, exposure to NO_2_ during the second trimester tended to present a slight association.

## Discussion

We examined the relationship between birth outcomes and exposure to air pollution in > 2,000 mother–child pairs in four different areas of Spain. We found that NO_2_ was statistically significant associated with birth length, with a decrease in length of around 1 mm in the combined analysis for each 10-µg/m^3^ increase in the whole pregnancy average for residential NO_2_ exposure. When the second trimester specifically was examined, the same increase in NO_2_ was associated with a nonsignificant reduction of 22 g in birth weight.

Our study presents a great deal of heterogeneity among the different exposure levels in the various cohorts, with higher NO_2_ levels in urban areas with greater traffic density (Sabadell and Valencia) and higher benzene levels in Asturias, which has more heavy industry than the other cohort areas. Although NO_2_ comes predominantly from vehicle traffic, benzene is additionally attributable to industrial emissions and residential heating emissions.

Because its primary source is traffic emissions, NO_2_ is considered to be a valid proxy for exposure to air pollution from traffic. Hence, it is one of the air pollutants most frequently assessed in relation to birth outcomes. However, comparisons between studies are difficult because of differences in exposure measurement methods and birth outcome definitions. We identified six studies similar to our study that analyzed the association between NO_2_ and birth weight as a continuous outcome. A significant association was found in three of them (Bell et al. 2007; Gouveia et al. 2004; Mannes et al. 2005), but not in the other three (Madsen et al. 2010; Salam et al. 2005; van den Hooven et al. 2009). In recent studies, several limitations have been noted by the authors, including misclassification of exposure assessment, lack of information on other types of exposure, and the importance of controlling for mobility or time–activity patterns. Our study attempted to address these issues as follows: *a*) The prospective design starting in early pregnancy made it possible to collect an extensive set of data on potential risk factors; *b*) the combined LUR approach, which included spatial and temporal information on NO_2_ distribution plus geographic information systems data, allowed us to estimate individual exposure indicators for each woman during various periods of pregnancy; *c*) a meticulous analysis controlled for risk factors and potential confounders; and *d*) taking changes in residence during pregnancy into account helped us avoid this source of misclassification. The association of NO_2_ exposure with birth length and weight was, in general, clearer in the subset of women who spent ≥ 15 hr/day at home. In fact, for average residential NO_2_ exposure, the estimated reduction in birth length in this subset was twice that of all the study subjects as a group. These results clearly show that time–activity patterns during pregnancy must be taken into account to improve the accuracy of exposure measurements and reduce exposure misclassification (Ritz and Wilhelm 2008). The magnitude of the association between NO_2_ and birth weight was similar to that found in two of the three studies in which a significant relationship was observed (Bell et al. 2007; Gouveia et al. 2004), but lower than that found in the Australian study (Mannes et al. 2005). The latter study found a greater magnitude for this relationship when the analysis was restricted to women who lived < 5 km from a monitoring station (Mannes et al. 2005). In our study the magnitude of association increased when we restricted our analysis to women who spent more hours at home. These findings suggest that when the assignation of exposure is improved, the association becomes clearer.

Still, our study has several limitations. First, information on other important pollutants associated with birth outcomes in previous studies was not available. However, as mentioned above, because NO_2_ is regarded as a marker of air pollution from vehicle traffic, it can be considered a good proxy for others pollutants, including particles (Clougherty et al. 2008). Second, because information regarding indoor pollution levels was unavailable, our exposure model is based only on outdoor levels. Although this could be considered a limitation, we were interested in assessing the effect of ambient environmental pollution, which we accomplished through this approach. Furthermore, in a paper recently published by our group, outdoor NO_2_ levels measured at 352 homes of women in the Valencia cohort proved to be an important predictor of indoor NO_2_ levels (Esplugues et al. 2010). Moreover, our models have been adjusted for exposure to environmental tobacco smoke and gas cooking, which are two important sources of indoor air pollution. Finally, because maternal smoking during pregnancy is known to affect fetal development, adjusting for this variable is crucial to avoid confounded estimates. Thus, we included maternal smoking during pregnancy in all the models analyzed, not only as a dichotomous variable, but also taking into consideration mothers that gave up smoking during first trimester. The estimates of the association with NO_2_ exposure did not change after including this variable.

Our results show a slight but significant association between a 10-µg/m^3^ increase in exposure to NO_2_ during pregnancy and birth length. This is especially interesting, as few studies have explored this relationship, probably because this anthropometric measure is not usually recorded in a standardized way. In a study based on information from a birth register in Brisbane, Australia (Hansen et al. 2007), maternal NO_2_ exposure during the third trimester was associated with a reduction in crown–heel length; similar associations were not found for other pollutants.

Several other studies examining pollutants other than NO_2_ have assessed prenatal exposure to air pollution and birth length. An international study including one cohort in Cracow, Poland, and another one in New York City (USA) (Choi et al. 2006) assessed the effects of airborne polycyclic aromatic hydrocarbon (PAH) exposure in the third trimester of pregnancy. In Cracow, where PAH levels were higher, such exposure was associated with a reduction in birth length, whereas no significant association between PAH and birth length was observed in the New York City cohort. This pattern persisted even when only women in the range of 1.80–36.47 ng/m^3^ of PAH exposure were considered (Choi et al. 2006).

Measuring birth length is a complex task, and some misclassification errors can be assumed. However, this potential misclassification would be nondifferential and probably unrelated to exposure levels at the residences of the women. To make sure, we analyzed whether our findings could be affected by the estimates of only one cohort, that is, if the association for birth length and NO_2_ exposure could be explained only by the association observed in Gipuzkoa. We found that excluding Gipuzkoa from the combined analysis did not substantially alter the association estimates for women spending ≥ 15 hr/day at home [a reduction of 0.15 cm (95% CI, –0.27 to –0.02 cm) vs. a reduction of 0.16 cm (95% CI, –0.27 to – 0.04 cm) for a 10-µg/m^3^ increase in NO_2_ throughout the pregnancy]. A 0.9-mm decrease in birth length may seem insignificant; however, after the distribution in our cohort, such a change would increase by 1.7% the risk of being small for gestational age for birth length.

We found no clear association between NO_2_ exposure and HC. Although the global estimate was not significant, a trend was found in the second trimester, which was significant for the Valencia cohort among women who spent more time at home. In the French EDEN (Etude des Déterminants pré et post natals du développement et de la santé de l’Enfant) cohort (Slama et al. 2006), a negative association between HC at birth and exposure to NO_2_ was found in the highest tertile (> 31.4 µg/m^3^) compared with the lowest. Further studies using this anthropometric measure are needed, especially because it is anatomically related with brain development.

Regarding the impact of benzene exposure in the French EDEN cohort (Slama et al. 2009), an association between personal maternal exposure in nonsmoking women and both head size and weight at birth was found. In our research, we found no significant associations. In a previous study conducted within the Sabadell cohort (Aguilera et al. 2009), an association was found between birth outcomes and exposure to the sum of benzene, toluene, ethyl-benzene, and xylene in women who spent < 2 hr/day in nonresidential outdoor environments. This finding suggests that volatile organic compounds other than benzene may have a negative impact on fetal growth that has yet to be elucidated.

Other studies have used multipollutant models to examine associations between pollution exposure and birth outcomes (Bell et al. 2007; Gouveia et al. 2004; Liu et al. 2007; Mannes et al. 2005; Salam et al. 2005; Wilhelm and Ritz 2005). For example, Liu et al. (2007) found that NO_2_ exposure during pregnancy was positively associated with fetal growth retardation, but the association disappeared when various pollutants were incorporated into the models. In contrast, Mannes et al. (2005), using multipollutant models, found NO_2_ to be the most important pollutant in the association between birth weight and air pollution. In our study, in which the correlation between NO_2_ and benzene is 0.44, the estimated effect of NO_2_ exposure on birth weight or length increased when benzene was included in the models.

Looking at critical exposure windows can provide insight into the biological mechanisms behind the impact of air pollution on fetal growth. Although the results have been inconsistent to date, the first months generally seem to be important (Bell et al. 2007; Mohorovic 2004). In our study the first and second trimester seemed to be the most relevant periods for NO_2_ exposure. NO_2_ is a potent oxidant, and elevated levels of methemoglobin, a marker of hemoglobin oxidation, have been found in the blood of mothers experiencing pregnancy complications (Tabacova et al. 1997). More recently, Mohorovic (2003) found similar methemoglobin results in a polluted area of Croatia. These findings suggest that maternal exposure to environmental oxidants early in pregnancy could increase the risk of pregnancy complications by stimulating the formation of methemoglobin, which may, in turn, lead to hypoxia and hypoxemia in pregnant women, affecting maternal health as well as placental and fetal development. Whether NO_2_ is simply a marker for other air pollutants from traffic or other types of combustion, as is the case with particulate matter or PAHs, is still undetermined. In our study, the association was clearer between NO_2_ exposure and a decrease in length than in weight. A mechanism that might explain such a differential effect has yet to be elucidated. Nevertheless, our results are in agreement with those described in a study conducted in Sweden examining the role of exposure to tobacco smoke in fetal development (Lindley et al. 2000). In that study the authors observed a differential association of tobacco smoke exposure with anthropometric measurements, in which the estimated effect was clearer for crown–heel length. In that study, the reduction in length also persisted in mothers who gave up smoking at early pregnancy, whereas this association disappeared for birth weight. Differential effects for tobacco smoke have been identified in animal experiments (Esposito et al. 2008), suggesting that the effect of exposure to toxins may be greater on length than on weight, because it is more related to bone development than to fetal volume. Further research is required to evaluate this phenomenon in relation to air pollution exposure.

Our findings contribute to the field of public health, because development problems in newborns may have health consequences later in adult life (Barker 2007). Moreover, the associations between exposure to air pollution from traffic during pregnancy and anthropometric measures observed in our study were observed at pollution levels similar to those recorded in urban areas around the world. This finding calls for the development of strategies to prevent exposure to harmful levels of air pollution during pregnancy.

## Conclusions

Our study is based on a prospective cohort with > 2,000 mother–child pairs. Air pollution exposure levels were estimated combining empirical measurements and geographic variables, whereas time–activity patterns were used to provide individual estimates of residential exposure to traffic-related air pollutants. In the combined analysis, among the subset of women who spent more time at home, we found a significant association between NO_2_ exposure and birth length in all three trimesters, taken both individually and for the entire pregnancy. There was a marginal association of birth weight with NO_2_ exposure in the second trimester of pregnancy. Benzene levels were not significantly associated with any particular outcome. Further research is needed to determine whether the associations observed are due to NO_2_ exposure itself or if this pollutant is simply a marker of other air pollution exposures. Given that these associations were found for pollution levels considered to be common in urban areas worldwide, strategies should be developed to reduce air pollution to prevent risks to fetal development.

## Supplemental Material

(296 KB) PDF
