# Supplementary material for: Residential Exposure to Outdoor Air Pollution during Pregnancy and Anthropometric Measures at Birth in a Multicenter Cohort in Spain
Source: Environ Health Perspect. 2011 Mar 23;119(9):1333–8. doi: 10.1289/ehp.1002918 (PMC3230392; doi:10.1289/ehp.1002918)
Supplement: (296 KB) PDF [file ehp.1002918.s001.pdf]

Supplemental material

Residential exposure to outdoor air pollution during pregnancy and anthropometric measures at birth in a multicenter cohort in Spain

Marisa Estarlich, Ferran Ballester, Inmaculada Aguilera, Ana Fernández-Somoano, Aitana Lertxundi, Sabrina Llop, Carmen Freire, Adonina Tardón, Mikel Basterrechea, Jordi Sunyer, and Carmen Iñiguez

Table of Contents

Table1: Description of the cohorts under study and their area of reference

Figure 1: Location of the four new INMA cohorts

Table 2: Data from each campaign and predictor variables of air pollution in each cohort

Table 3: Characteristics of the women participating in the study by cohort

Supplemental material, Table 1: Description of the cohorts under study and their area of reference

Cohort	Region	Extension (km^2) of the area	Number of municipalities	Reference population	Description	Recruited pregnant women (early pregnancy)	Study population (at delivery)
Asturias	Asturias	483	9	160000	Included one urban zone, towns in a semi-urban area and rural municipalities. It is a typical industrial zone.	494	417
Gipuzkoa	Basque Country	519	26	90000	The area is divided into three narrow valleys that have a high grade of unevenness. Metallurgy is the principal industrial activity.	638	573
Sabadell	Catalonia	38	1	200000	Composed of a mainly urban area.	657	563
Valencia	Valencian Community	1372	32	300000	Composed of a typically urban zone (city of Valencia), a metropolitan area, a semi-urban mixed area including residential and industrial zones as well as an agricultural area, and a typically rural zone.	855	784

Supplemental material, Figure 1: Location of the four new INMA cohorts

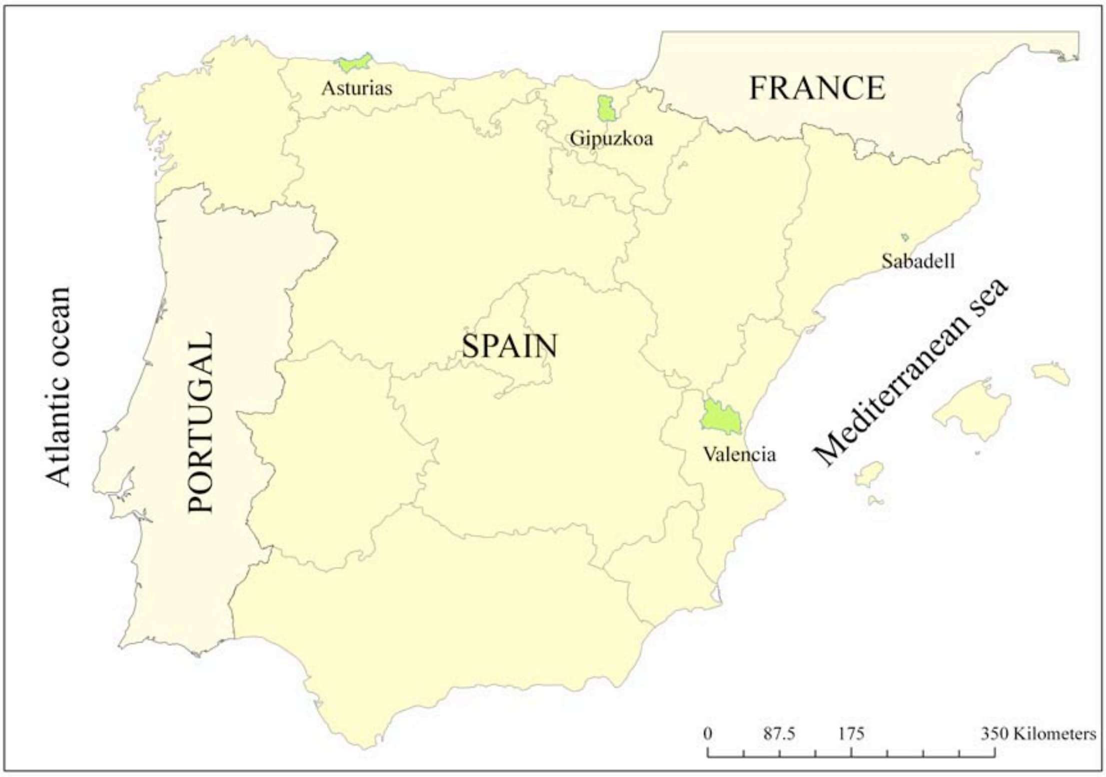

Supplemental material, Table 2a: Data from each campaign and predictor variables of air pollution in each cohort

NO_2

Cohort	no. passive samplers	Date campaigns	Predictor variables	R^2 model	Number of monitoring stations	Pollutant used to correct for seasonality
Asturias	67	June 05<br>November 05	Altitude<br>Distance to nearest road [a]<br>Agricultural or forest land cover (300 m-buffer)	0.521	4	NO_2
Gipuzkoa	85	February 07<br>June 07	Altitude (3 cat)<br>Valley factor<br>Distance to nearest road [a] (MDI[b]>20000)<br>Urban land cover (100 m-buffer)<br>Industrial land cover (300 m-buffer)	0.509	3	NO_2
Sabadell	57	April 05<br>June 05<br>October 05<br>March 06	Altitude<br>Urban or industrial land cover (500 m-buffer)<br>Road type (minor, major, secondary road)	0.750	1	NO_2
Valencia	93	April 04<br>June 04<br>November 04<br>February 05	Kriging[c]<br>Industrial or urban land cover (500 m-buffer)<br>Distance to nearest major road[a] (MDI[b]>10000)	0.730	7	NO_2

[a] Distance to the nearest major road (in logarithms)

[b] MDI: Mean daily traffic count

[c] Mean of estimated NO_2 from kriging among campaigns

Supplemental material, Table 2b: Data from each campaign and predictor variables of air pollution in each cohort

BENZENE

Cohort	no. passive samplers	Date campaigns	Predictor variables	R^2 model	Number of monitoring stations	Pollutant used to correct for seasonality	Correlation with benzene
Asturias	67	June 05<br>November 05	Altitude<br>Distance to nearest major road (MDI[b] 1001-5000)<br>Continuous urban land cover (300 m-buffer)<br>Discontinuous urban land cover (1000 m-buffer)<br>Agricultural or forest land cover (1000 m-buffer)	0.728	4	SO_2	0.30
Gipuzkoa	85	February 07<br>June 07	Valley factor<br>Distance to nearest road [a] (MDI[b]>20000)<br>Urban land cover (100 m-buffer)<br>Distance to industry	0.437	3	NO_2	0.80
Sabadell	57	April 05<br>October 05<br>March 06	Road type (High/medium/low traffic)<br>Inhabitants (50 m-buffer)<br>Urban land cover (300 m-buffer)<br>Number of buildings (500 m-buffer)	0.724	1	NO_2	0.40
Valencia	93	April 04<br>June 04<br>February 05	Urban land cover (500 m-buffer)<br>Distance to nearest road [a] (MDI[b]>50000)<br>Longitude	0.437	7	NO	0.40

[a] Distance to the nearest major road (in logarithms)

[b] MDI: Mean daily traffic count

Supplemental material, Table 3a: Characteristics of the women participating in the study by cohort.

Characteristics N (%)	Study population					
	Overall	Asturias	Gipuzkoa	Sabadell	Valencia	
	2337	417	573	563	784	p[a]
Newborn						
Sex, boy	1213 (51.9)	224 (53.7)	290 (50.6)	285 (50.6)	414 (52.8)	0.669
Birth weight, grams. Mean (SD)	3342.0 (400.6)	3364.9 (386.1)	3367.3 (392.3)	3311.7 (391.0)	3333.2 (419.4)	0.065
Birth length, cm. Mean (SD)	49.9 (1.8)	50.1 (1.9)	49.2 (1.7)	49.6 (1.7)	50.5 (1.8)	<0.001
Birth head circumference, cm. Mean (SD)	34.4 (1.3)	34.4 (1.3)	34.8 (1.3)	34.3 (1.1)	34.3 (1.3)	<0.001
Mother						
Age, years. Mean (SD)	30.6 (4.3)	31.5 (4.5)	31.4 (3.6)	30.2 (4.3)	29.8 (4.5)	<0.001
Height, cm. Mean(SD)	162.6 (6.2)	162.0 (6.1)	164.1 (5.9)	162.4 (6.1)	162.1 (6.4)	<0.001
Pre-pregnancy weight						0.274
<50	174 (7.4)	34 (8.2)	39 (6.8)	44 (7.8)	57 (7.2)	
50-59	925 (39.6)	156 (37.4)	232 (40.5)	225 (39.9)	312 (39.8)	
60-69	753 (32.2)	141 (33.8)	204 (35.6)	173 (30.7)	235 (29.9)	
>69	485 (20.8)	86 (20.6)	98 (17.1)	121 (21.5)	180 (22.9)	
Rate of weight gain during pregnancy						0.040
Low	547 (23.4)	103 (25.3)	129 (24.1)	116 (21.3)	199 (25.3)	
Recommended	856 (36.6)	152 (37.3)	229 (42.7)	205 (37.6)	270 (34.8)	
High	860 (36.8)	152 (37.3)	178 (33.2)	224 (41.1)	306 (39.5)	
Education						<0.001
Primary or less	586 (25.1)	80 (19.2)	77 (13.5)	163 (29.1)	266 (33.9)	
Secondary	955 (40.9)	187 (44.8)	199 (34.9)	236 (42.1)	333 (42.5)	
University	791 (33.8)	150 (36.0)	295 (51.7)	161 (28.8)	185 (23.6)	
Social class						<0.001
I, II	490 (21.0)	86 (20.7)	170 (29.7)	111 (19.7)	123 (15.7)	
III	599 (25.6)	85 (20.4)	162 (28.3)	166 (29.5)	186 (23.7)	
IV, V	1247 (53.4)	245 (58.9)	241 (42.1)	286 (50.8)	475 (60.6)	
Working during pregnancy (1st trimester)	1623 (69.1)	254 (60.2)	451 (77.8)	435 (77.3)	483 (61.6)	<0.001
Working during pregnancy (3rd trimester)	766 (32.6)	103 (24.4)	167 (28.8)	215 (38.2)	281 (35.8)	<0.001
Country of origin: Spain	2135 (91.4)	402 (96.4)	550 (96.0)	492 (88.7)	691 (88.1)	<0.001
Living with the father	2295 (98.2)	408 (97.8)	570 (99.5)	555 (98.8)	762 (97.2)	0.010
Nulliparous	1022 (43.7)	254 (60.9)	310 (54.1)	319 (56.9)	430 (54.8)	0.141
Smoking during pregnancy (still at 3rd trimester)	385 (16.5)	67 (17.1)	64 (11.3)	79 (14.3)	177 (22.8)	<0.001
Passive smoking at home	772 (33.0)	124 (31.6)	62 (11.1)	201 (36.4)	365 (47.2)	<0.001
Global passive smoking	1509 (64.6)	217 (52.0)	340 (59.3)	367 (65.2)	585 (74.6)	<0.001
Paternal height, cm. Mean(SD)	175.9 (6.9)	175.1 (6.8)	177.1 (6.5)	175.9 (7.1)	175.6 (7.2)	<0.001

[a] : p- value for the comparison among cohorts. ANOVA for continuous variables and X^2 test for categorical ones

Supplemental material, Table 3b (cont. table 3a): Characteristics of the women participating in the study by cohort.

Characteristics N (%)	Study population					
	Overall	Asturias	Gipuzkoa	Sabadell	Valencia	
	2337	417	573	563	784	p[a]
Season of delivery						<0.001
Winter	614 (26.3)	96 (23.0)	170 (29.7)	151 (26.8)	197 (25.1)	
Spring	582 (24.9)	81 (19.4)	169 (29.5)	170 (30.2)	162 (20.7)	
Summer	544 (23.3)	121 (29.0)	132 (23.0)	129 (22.9)	162 (20.7)	
Autumn	597 (25.5)	119 (28.5)	102 (17.8)	113 (20.1)	263 (33.5)	
Type of cooker						<0.001
Electric	1236 (54.3)	304 (77.6)	468 (83.9)	202 (36.6)	262 (33.8)	
Gas	1024 (45.0)	85 (21.7)	86 (15.4)	345 (62.5)	508 (65.5)	
Others	18 (0.8)	3 (0.8)	4 (0.7)	5 (0.9)	6 (0.8)	
Type of zone						<0.001
Urban	2241 (95.9)	400 (95.9)	542 (94.6)	563 (100)	736 (93.9)	
Rural	96 (4.1)	17 (4.1)	31 (5.4)	b	48 (6.1)	
Women who spent more than 15 hours at home	1380 (59.1)	356 (91.0)	233 (42.0)	297 (54.0)	494 (63.7)	<0.001
Levels of air pollution						
NO_2 Mean (SD)						
All	29.2 (11.1)	23.5 (6.5)	20.1 (6.4)	31.9 (8.6)	36.9 (11.1)	<0.001
Urban	29.8 (11.0)	23.8 (6.5)	20.2 (6.4)	31.9 (8.6)	38.3 (9.8)	
Rural	16.4 (4.9)	18.0 (5.3)	17.5 (5.9)	b	15.1 (3.9)	
Benzene Mean (SD)						
All	1.6 (1.1)	2.3 (1.3)	1.0 (0.3)	0.81 (0.3)	2.17 (0.6)	<0.001
Urban	1.6 (0.9)	2.3 (1.3)	1.0 (0.3)	0.81 (0.3)	2.2 (0.6)	
Rural	1.5 (0.7)	1.7 (1.1)	0.9 (0.2)	b	1.7 (0.6)	

[a] : p- value for the comparison among cohorts. ANOVA for continuous variables and X^2 test for categorical ones
na: not applicable
